# Application of Antibody and Immunoassay for Food Safety

**DOI:** 10.3390/foods11060826

**Published:** 2022-03-14

**Authors:** Hongtao Lei, Zhanhui Wang, Sergei A. Eremin, Zhiwei Liu

**Affiliations:** 1 Guangdong Province Key Laboratory of Food Quality and Safety/National-Local Joint Engineering Research Center for Machining and Safety of Livestock and Poultry Products, College of Food Science, South China Agricultural University, Guangzhou 510642, China; liuzhiwei2021888@163.com; 2 Beijing Advanced Innovation Center for Food Nutrition and Human Health, College of Veterinary Medicine, China Agricultural University, Beijing 100193, China; wangzhanhui@cau.edu.cn; 3 Beijing Key Laboratory of Detection Technology for Animal-Derived Food Safety, Beijing Laboratory for Food Quality and Safety, Beijing 100193, China; 4 Department of Chemical Enzymology, Faculty of Chemistry, M.V. Lomonosov Moscow State University, 119991 Moscow, Russia; eremin_sergei@hotmail.com

This Special Issue of Foods, Application of Antibody and Immunoassay for Food Safety, contains ten papers that were refereed and selected in accordance with the usual editorial standards of the journal.

The aim of this Special Issue is to advance the current state of knowledge concerning antibodies and immunoassays for the detection of chemical and biological analytes such as food contaminants, food fraud, and so on, in the field of food safety.

Food safety is of critical societal importance for producers, food agencies, regulatory bodies and consumers. Therefore, there is a need to develop fast, sensitive, reliable, cost-effective, and easy-to-use analytical techniques for the protection of food safety and quality. Currently, instrumental analysis methods are commonly used for food safety purposes, such as high-performance liquid chromatography (HPLC), high-performance liquid chromatography–tandem mass spectrometry (HPLC-MS/MS), etc. Although the aforementioned assays are validated, sensitive, and reliable, they are unsuitable for application in rapid screening and field detection owing to the requirements of expensive apparatus, time-consuming operation, and highly skilled personnel. Immunoassays, a class of analytical techniques based on the specific recognition between antibody and antigen, are preferable to overcome these obstacles because of their high sensitivity, specificity, rapidity and cost-effectiveness, which allow them to play a prominent role in the rapid detection of various analytes in food safety [1].

To pursue higher sensitivity, advances have been made to improve the analytical sensitivity of immunoassays. In particular, chemiluminescent immunoassay (CLEIA) and fluoroimmunoassay (FIA) are two commonly proposed methods to meet the needs of strict screening. Ou et al. [2] prepared a monoclonal antibody against aristolochic acid I (AA-I) and applied it in CLEIA and FIA for the highly sensitive determination of aristolochic acid I (AA-I) in foods (slimming capsule, slimming tea, and pleurotus ostreatus). The proposed CLEIA showed higher sensitivity compared with conventional ELISA. On the other hand, a novel fluorescent probe, carbon dots, was synthesized and employed in FIA, which exhibited a five-fold greater enhancement in sensitivity than CLEIA. Moreover, the accuracy and practicability of CLEIA and FIA were verified by the standard instrument method, indicating that both were sensitive, rapid, and easy to use, making them effective tools for screening AA-I in related products. Additionally, there are also various emergent strategies that address the poor sensitivity of immunoassays, including novel signal labels (i.e., nanozymes and magnetic-loaded nanoparticles), unique antibody with unique nature, and heterologous strategies adjusting the binding capability of the competitive antigen, as well as in combination with innovative detection platform (i.e., microfluidic detection platform, smart detection systems, and a detection platform combined with molecular biology).

Despite enormous sensitivity-enhanced strategies for immunoassays, the unwanted interference from food matrix is still a major factor affecting assay sensitivity due to the complexity and variability of matrix compounds in food samples, which might greatly affect the immunological reaction. Assessing the matrix interference on the assay sensitivity is thus an important issue in the development of methods. Burkin et al. [3] firstly evaluated the influence of avidin (AVI) and biotin (B7) contained in food matrices on two kinds of (Strept)avidin–biotin-based enzyme-linked immunosorbent assays (ELISAs) for bacitracin (BT) and colistin (COL) determination, with simultaneous assessment of extraneous AVI/B7 and AVI/B7 from different matrices (egg, infant milk formulas enriched with B7, and chicken and beef liver). Summarizing the experience of the present study, it is recommended to avoid immunoassays based on avidin–biotin interactions when analyzing samples containing these endogenous factors or enriched with B7.

Immunoassays, especially for ELISA, are generally heterogeneous, and involve repeated washing and a certain degree of reaction time. In contrast, fluorescence polarization immunoassay (FPIA) is a homogeneous assay format without separation or washing, giving the advantages of rapidity, reliability, and ease of use. It is based on the competition between an analyte and a fluorescein-labeled tracer for binding antibody. Zhang et al. [4] established an FPIA for 4,4′-dinitrocarbanilide (DNC) in chicken samples, with favorable sensitivity, specificity, cost, time, and reliability. The sensitivity of the developed FPIA was significantly improved by optimizing the selection of 25 tracers, tracer–antibody pairs, and physical and chemical reaction conditions. Furthermore, the reliability and robustness of the assay were successfully demonstrated for the analysis of DNC in chicken muscle matrices. The total analysis time, including sample pretreatment, was less than 40 min, which has not yet been achieved in other immunoassays for DNC. Up to now, many FPIA for other analytes such as mycotoxins, pesticides, antibiotics, and so on, have been tested and compared favorably with instrumental reference methods.

Compared with the ELISA-based and FPIA assays mentioned above, another immunoassay, namely lateral flow immunochromatography assay (LFIA), has gained increasing popularity because of its simple operation, rapidity, sensitivity, and cost-effectiveness. Li et al. [5] focus on the development of a rapid, convenient and sensitive LFIA based on traditional Au nanoparticles (AuNPs) for furosemide in slimming health foods, and the results could be read by the naked eye within 12 min (including sample pretreatment). The qualitative limit of detection (LOD) of the AuNPs-LFIA was 1.0–1.2 μg/g in slimming health foods. The developed method showed high consistency with liquid chromatography–tandem mass spectrometry (LC-MS/MS), and no false positive or false negative results were found in spiked slimming health foods. However, AuNPs-LFIA is known to have limited sensitivity because of AuNPs’ narrow particle size range and poor colloid stability. Wu et al. [6] described a background fluorescence-quenching immunochromatographic assay (bFQICA) in which AuNPs were used to quench the fluorescence of a background fluorescence baseboard instead of using the colorimetric method. Such a method was optimized, validated, and applied in the rapid on-site detection of nitrofurazone metabolite of semicarbazide (SEM) residues in animal-derived foods (egg, chicken, fish, and shrimp). Indeed, compared with the traditional AuNP-LFIA method, the detectability of the bFQICA method was higher, and the detection time was shortened compared with heterogeneous reactions such as ELISA. In addition, the quantitative results of SEM can be directly displayed by using a portable fluorescence immunoquantitative analyzer and a QR code with a built-in standard curve, which is efficient and convenient. Additionally, the signal label is a vital factor in the performance of LFIA. Novel nanoparticle labels have been introduced to obtain satisfactory sensitivity. Chen et al. [7] designed a chiral carbon containing a structure similar to that of propiconazole, and a polyclonal antibody that specifically recognizes propiconazole was obtained for the first time. Based on this antibody, a time-resolved fluorescence microspheres lateral flow immunochromatographic assay (TRFMs-LFIA) was developed, optimized, and evaluated for its sensitivity, specificity, and recovery. The analysis of blind real-life samples (brassica campestris, lettuce, and romaine lettuce) showed a good agreement with results obtained using HPLC-MS/MS. Of course, there are many other nanoparticle labels have been synthesized for the enhancement of LFIA performance. Despite these advances, some still need to be improved in order to enhance their high-throughput capacity in a single assay, and to move toward miniaturization involves the use of mobile devices such as smartphones.

Some scholars have also focused on preparing specific recognition molecules with unique performance, such as broad specificity, low molecule weight or small size (single-chain variable fragment (ScFv)), disulfide-stabilized antibodies, antigen-binding fragment (Fab), nanobodies, bispecific monoclonal antibodies (BsMAbs), aptamers, molecularly imprinted polymers, etc. In recent years, BsAbs, broad-spectrum antibodies, and ScFv have been increasingly favored by researchers in the field of immunoassays.

Compared with a single-specific antibody which can only recognize one antigen in a complex food matrix, a bispecific monoclonal antibody (BsMAb) with two distinct antigen-binding sites could recognize two different target analyses, which is more efficient, convenient, and economical. Lu et al. [8] successfully prepared BsMAb against aflatoxin B1 (AFB1) and ochratoxin A (OTA), and developed a novel and efficient immunoaffinity column (IAC) based on BsMAb for the rapid and effective extraction of AFB1 and OTA with a one-time extraction from corn and wheat samples. Then, the ELISA for AFB1 and OTA were applied, combined with IAC, with a satisfactory matrix effect elimination effect and recovery rate. The development of BsMAb has opened a whole new field in multi-analyte detection. Future advances will include, but not be limited to, exploiting new methods based on BsMAb, and novel techniques of antibody development that will allow for two or more targets, as well as cheaper and faster analysis methods.

An immunoassay based on a broadly specific antibody is an emerging trend in the sensitive and simple detection of a group of similar compounds in a single assay. Shao et al. [9] designed and synthesized an unreported hapten, 5-(propylthio)-1H-benzo[d]imidazol-2-amine, which maximally exposed the characteristic sulfanyl group of albendazole (ABZ) to the animal immune system to induce the expected antibody. One mAb that can simultaneously detect the sum of ABZs (ABZ and its metabolites, i.e., ABZSO_2_, ABZSO, and ABZNH_2_SO_2_) was obtained. The results of computational chemistry methods revealed that the hydrophobicity and conformation of a characteristic group of molecules might be the key factors that together influence the antibody recognition of these analytes. Furthermore, the practicability of the developed ELISA was verified by detecting ABZs in spiked milk, beef, and liver samples with recoveries. Zhang et al. [10] explored and developed novel haptens using molecular modeling to prepare broad-spectrum mAbs against brevetoxin 2 (PbTx-2), 1 (PbTx-1), and 3 (PbTx-3), followed by an ELISA method to detect brevetoxins in oyster samples was developed. In particular, the differences between the haptens of PbTx-2-CMO and PbTx-2-HS were evaluated using molecule alignment and electrostatic potential analysis. The results highlight that the spacer HS arm of PbTx-2-HS formed a specific spatial conformation with the parent nucleus structure, non-conducive to the production of high-affinity antibodies against the target, while PbTx-2-CMO was the ideal hapten to be used for antibody production due to its similar structure to the target, which was also further verified by antibody production and characterization. Besides ideal specificity and recovery rate, the sensitivity of the proposed ELISA based on such a mAb was higher than that of the high-resolution LC-MS, providing a useful method for monitoring PbTxs in oyster samples. The two studies above revealed that hapten design is an important feature when preparing antibodies against multiple target compounds. Importantly, molecular modeling and theoretical tools may assist immunochemists to find the most appropriate hapten chemical structure for broad-spectrum antibody production.

Single-chain variable fragments (scFv), as one of the most common formats of recombinant antibody, possess only one chain of the complete antibody while maintaining antigen-specific binding abilities, and can be expressed in a prokaryotic system. Moreover, it can be easily engineered with enhanced affinity and selectivity. Wang et al. [11] constructed an immunized mouse phage display single-chain variable fragment (scFv) library for the screening of recombinant anti-ciprofloxacin single-chain antibody for the detection of ciprofloxacin (CIP) in animal-derived food. The highest positive scFv-22 was expressed in *E. coli* BL21. Specifically, its recognition mechanisms were studied using the molecular docking method, and directional mutagenesis was performed for sensitivity improvement. The results of the established icELISA demonstrate that the ScFv mutant showed 16.6-fold improved sensitivity compared with parental scFv. Although scFvs have already found widespread use in clinical therapy and imaging procedures in the past decades, the use of such antibody fragments can provide clear benefits in terms of assay performance and relatively easy preparation in comparison to monoclonal and polyclonal antibodies, which will most probably lead to the increased use of recombinant antibodies in analytical applications in the near future.

To conclude, the present Special Issue addresses several critical issues, ranging from methodologies for performance enhancement (sensitivity, rapidity, and reliability), the assessment of food matrixes, and the development of specific recognition elements (BsMAb, broad-spectrum antibodies, and ScFv mutants). To satisfy higher detection requirements, the development of ultrasensitive and accurate immunoassays with multiple detection abilities is a growing trend. Exploiting high-quality antibodies and new specific recognition elements is particularly important to assay performance. Additionally, preparing stable and strong signal labels is necessary for improving the accuracy and sensitivity of immunoassays. Moreover, multiplex testing technologies are also inevitable trends in the development of immunoassays.

Finally, we thank the authors for their valuable contributions to this Special Issue.

## References

[1] Zhang H., Li B., Liu Y., Chuan H., Liu Y., Xie P. (2022). Immunoassay technology: Research progress in microcystin-LR detection in water samples. J. Hazard. Mater..

[2] Ou A.-F., Chen Z.-J., Zhang Y.-F., He Q.-Y., Xu Z.-L., Zhao S.-Q. (2021). Preparation of Anti-Aristolochic Acid I Monoclonal Antibody and Development of Chemiluminescent Immunoassay and Carbon Dot-Based Fluoroimmunoassay for Sensitive Detection of Aristolochic Acid I. Foods.

[3] Burkin M.A., Galvidis I.A., Eremin S.A. (2022). Influence of Endogenous Factors of Food Matrices on Avidin–Biotin Immunoassays for the Detection of Bacitracin and Colistin in Food. Foods.

[4] Zhang Q., Zou M., Wang W., Li J., Liang X. (2021). Design, Synthesis, and Characterization of Tracers and Development of a Fluorescence Polarization Immunoassay for Rapid Screening of 4, 4′-Dinitrocarbanilide in Chicken Muscle. Foods.

[5] Li Y.Y., Xie H.H., Wang J., Li X.M., Xiao Z.L., Xu Z.L., Lei H.T., Shen X. (2021). Lateral Flow Immunochromatography Assay for Detection of Furosemide in Slimming Health Foods. Foods.

[6] Wu Y.P., Wang J., Zhou Y., Qi Y.H., Ma L.C., Wang X.N.A., Tao X.Q. (2021). Quantitative Determination of Nitrofurazone Metabolites in Animal-Derived Foods Based on a Background Fluorescence Quenching Immunochromatographic Assay. Foods.

[7] Chen B., Shen X., Li Z., Wang J., Li X., Xu Z., Shen Y., Lei Y., Huang X., Wang X. (2022). Antibody Generation and Rapid Immunochromatography Using Time-Resolved Fluorescence Microspheres for Propiconazole: Fungicide Abused as Growth Regulator in Vegetable. Foods.

[8] Lu D., Wang X., Su R., Cheng Y., Wang H., Luo L., Xiao Z. (2022). Preparation of an Immunoaffinity Column Based on Bispecific Monoclonal Antibody for Aflatoxin B1 and Ochratoxin A Detection Combined with ic-ELISA. Foods.

[9] Shao S., Zhou X., Dou L., Bai Y., Mi J., Yu W., Zhang S., Wang Z., Wen K. (2021). Hapten Synthesis and Monoclonal Antibody Preparation for Simultaneous Detection of Albendazole and Its Metabolites in Animal-Origin Food. Foods.

[10] Zhang X., Ding M., Zhang C., Mao Y., Wang Y., Li P., Jiang H., Wang Z., Yu X. (2021). Development of a New Monoclonal Antibody against Brevetoxins in Oyster Samples Based on the Indirect Competitive Enzyme-Linked Immunosorbent Assay. Foods.

[11] Wang F., Li N., Zhang Y., Sun X., Hu M., Zhao Y., Fan J. (2021). Preparation and Directed Evolution of Anti-Ciprofloxacin ScFv for Immunoassay in Animal-Derived Food. Foods.

